# Anti-inflammatory effects of empagliflozin in patients with type 2 diabetes and insulin resistance

**DOI:** 10.1186/s13098-018-0395-5

**Published:** 2018-12-18

**Authors:** Sachiko Hattori

**Affiliations:** Department of Endocrinology and Metabolism, Tohto Clinic, 4-1, Kioi-Cho, Chiyoda-Ku, Tokyo, 102-0094 Japan

**Keywords:** Sodium-glucose co-transporter-2 inhibitor, High sensitivity CRP, Insulin resistance

## Abstract

**Background:**

Inflammation might be a pathological mediator of cardiovascular events in patients with type 2 diabetes and high cardiovascular risk.

**Methods:**

We investigated whether empagliflozin (EMPA) exerts anti-inflammatory effects that are reflected in decreased high-sensitivity C-reactive protein (hsCRP) values. Patients were allocated to receive a placebo (n = 51) or EMPA (n = 51) as an add-on treatment. Fasting blood samples were collected before and every 3 months after this intervention for 1 year.

**Results:**

Empagliflozin tended to elicit reductions in BMI, HbA1c, aspartate aminotransferase, alanine aminotransferase (ALT), and gamma-glutamyl transpeptidase compared with the placebo, but the differences did not reach statistical significance. Levels of LDL-cholesterol, HDL-cholesterol, and triglycerides were unaltered, significantly increased, and decreased, respectively, by EMPA, but the differences were not statistically significant compared with the placebo. Empagliflozin for 12 months notably reduced the homeostatic model assessment of insulin resistance (HOMA-IR), remnant-like particle cholesterol (RLP-C), and hsCRP by 43%, 52% and 54%, respectively. The time courses of these reductions significantly differed from those of the placebo. Systolic and diastolic blood pressure were also significantly reduced by EMPA compared with the placebo. We applied multiple linear regression analysis to determine which factors were associated with changes in hsCRP induced by EMPA. The results revealed that alterations in hsCRP values (log [hsCRP at 12 months] minus log [hsCRP at month 0]) were significantly associated with changes in HOMA-IR, RLP-C, systolic blood pressure, HDL-C and ALT.

**Conclusion:**

Empagliflozin decreased hs-CRP and lowered levels of remnant related lipoproteins probably via ameliorating insulin resistance. The cardiovascular benefits conferred by EMPA might be driven at least partly by anti-inflammatory effects, and this mechanism might cooperate with other EMPA-induced changes including reduced blood pressure, to achieve the degree of cardioprotection revealed by the EMPA-REG OUTCOME trial.

*Trial registration* UMIN Clinical Registry (UMIN000021552). Registered 21 March 2016, https://upload.umin.ac.jp/UMIN000021552

## Background

The sodium–glucose cotransporter 2 inhibitor, empagliflozin (EMPA) reduced relative risk for cardiovascular (CV) mortality, hospitalization for heart failure and death from any cause by 38%, 35% and 32%, respectively, in the EMPA-REG OUTCOME trial of patients with type 2 diabetes and high risk of CV [1]. The results of a sub-analysis of the EMPA-REG OUTCOME study of Asian patients with type 2 diabetes and high CV risk were also striking [2]. Thus, the EMPA-REG OUTCOME trial established that EMPA is cardioprotective in high-risk patients with diabetes, but the underlying mechanisms remain elusive.

Obesity-related, subacute chronic inflammation is associated with incident type 2 diabetes and atherosclerotic CV disease. Inflammation might be a pathological mediator of these commonly concurrent pathologies. Ridker et al. [3] recently showed that anti-inflammatory therapy with canakinumab, which targets the interleukin-1β innate immunity pathway, leads to a significantly lower rate of recurrent CV events than a placebo, independently of lowering LDL-cholesterol. They showed that the magnitude of the reduction in high-sensitivity C-reactive protein (hsCRP) after a single dose of canakinumab might provide a simple clinical method with which to identify individuals who are likely to benefit from continued treatment; that is, lower is better for reducing inflammation with canakinumab [4].

Empagliflozin exerts anti-inflammatory effects in experimental animal models [5, 6], but not in humans. Here, we aimed to determine whether EMPA exerts anti-inflammatory effects and whether the CV benefits of EMPA are due partly to such effects, implied by decreased hsCRP values in patients with type 2 diabetes and insulin resistance.

## Methods

### Study design and participants

This single-center, open-label, randomized, prospective study enrolled patients without a history of medication with SGLT2 inhibitors and with HbA1c > 6.2% regardless of diet, exercise, and medical treatment other than SGLT2 inhibitors for at least 12 weeks. They were assessed at our clinic and might have had insulin resistance (BMI > 28 or homeostatic model assessment of insulin resistance [HOMA-IR] > 1.73 or fasting immunoreactive insulin [IRI] > 10 and fasting blood glucose [FBG] < 180). The patients were then allocated to receive EMPA (10 mg; n = 51) or a placebo (n = 51) as an add-on treatment. All patients continued with their administered oral hypoglycemic drugs (sulfonylureas, metformin, or an α-glucosidase inhibitor), antihypertensive agents (angiotensin II receptor blockers or calcium channel blockers), and antihyperlipidemic agents (statins or fibrates) (Table 1).

**Table 1:** Baseline charasteristics and medicatoins of the participants

	Placebo (n = 51)	EMPA (n = 51)	P value
Age (years)	58.1 ± 9.71	57.4 ± 12.3	0.778
Male/(female)	41 (10)	38 (13)	0.636
Baseline medication			
Slfonylureas	11	10	1
Metformin	12	12	1
α-GI	8	7	1
ARB	16	17	0.915
CCB	10	10	1
Statins	17	16	1
Fibrates	9	10	0.879

Data were expressed as mean ± standard deviation

*α*-*GI* α-glicosidase inhibitor, *ARB* angiotensin II receptor blocker, *CCB* calcium channel blocker

### Measurements

Overnight fasting blood and urine samples were obtained at baseline and after every 3 months of EMPA or placebo administration for 1 year. All biochemical data were obtained at our laboratory, except IRI, remnant-like particle cholesterol (RLP-C), hsCRP and urinary albumin, which were assessed at LSI Medicine Corporation (Tokyo, Japan). We calculated HOMA-IR every 3 months as follows: EMPA was stopped for 72 h until urinary glucose was undetectable and then blood values of fasting glucose and IRI were measured.

### Statistical analysis

Data are expressed as mean ± standard deviation (SD). Data were statistically analyzed using EZR software version 1.21 [7]. Parameters before and 3, 6, 9, and 12 months after treatment were compared using paired t-tests. Changes in parameters over a period of 12 months between placebo and EMPA were analyzed using repeated measures ANOVA. Factors associated with changes in hsCRP were determined using multiple linear regression analysis. Continuous and categorical baseline values and medications were analyzed using t-tests and Fisher exact tests, respectively. Differences were considered statistically significant at p < 0.05.

## Results

**Fig. 1:**
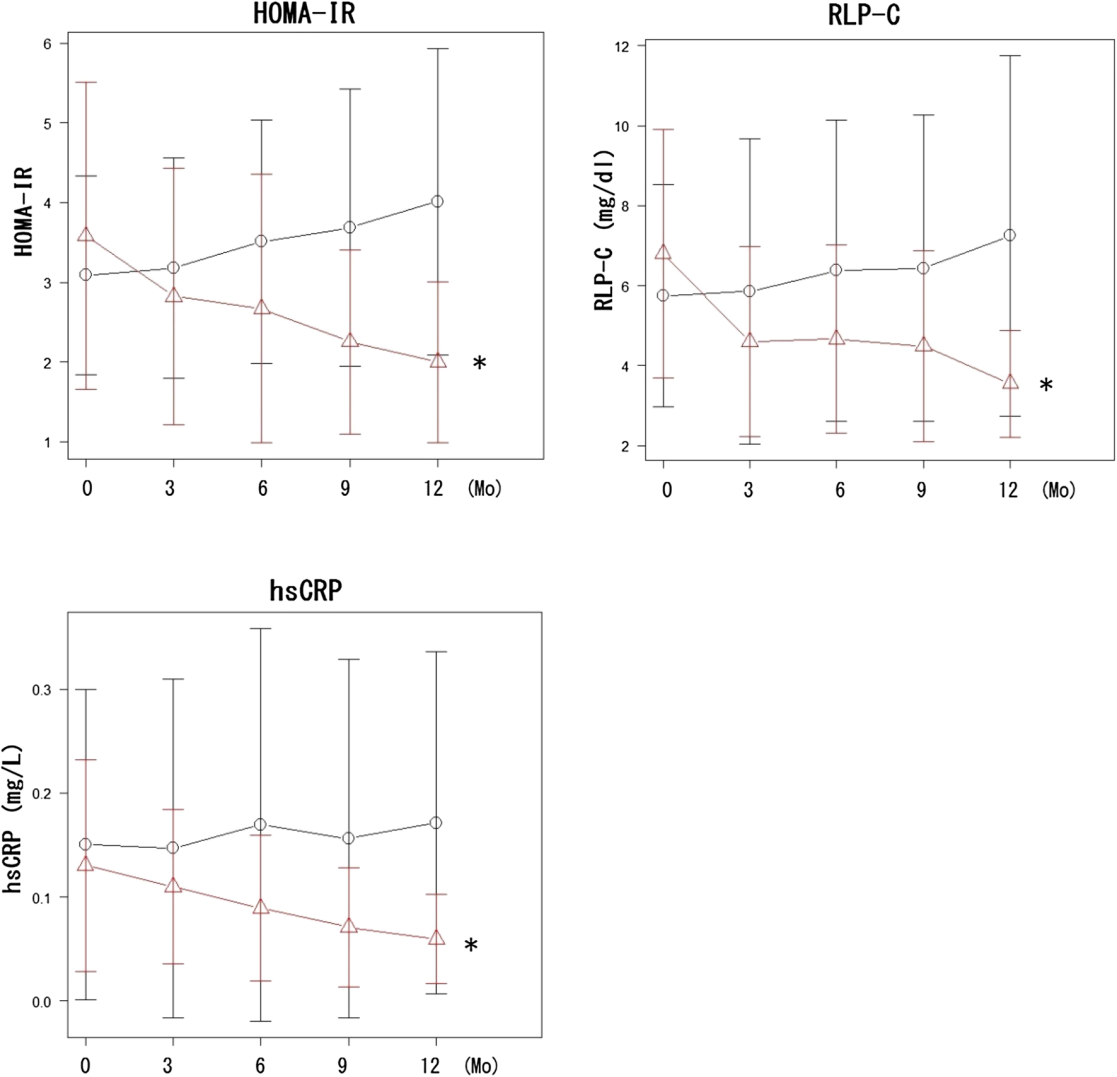
Time course of HOMA-IR, RLP-C and log[hsCRP] in patients treated with EMPA (triangles, red line) or placebo (circles, black line). Data are presented as mean ± SD. Group differences parameters between baseline and 12 months later between placebo and EMPA were analyzed *p < 0.05 (repeated measures ANOVA); p = 0.0163, p = 0.029 and p = 0.00706 for HOMA-IR, RLP-C and hsCRP, respectively

Levels of BMI, HbA1c, aspartate aminotransferase (AST), alanine aminotransferase (ALT) and gamma-glutamyl transpeptidase (γ-GTP) were slightly but not significantly reduced by EMPA compared with the placebo. Levels of LDL-cholesterol, HDL-cholesterol, and triglycerides were unaltered, significantly increased, and reduced, respectively, by EMPA, but the differences did not reach statistical significance compared with the placebo. Notably, HOMA-IR, RLP-C, and hsCRP were reduced by 43%, 52%, and 54%, respectively, after 12 months of EMPA. The time courses of these reductions significantly differed between EMPA and the placebo. Systolic (SBP) and diastolic blood pressure (DBP) were significantly reduced by EMPA, but not by the placebo. Estimated glomerular filtration rates (eGFR), urinary albumin excretion (measured as urinary albumin-to-creatinine ratios; ACR) did not significantly differ between the placebo and EMPA groups (Table 2, Fig. 1).

**Table 2:** Clinical parameters of patients treated with placebo or EMPA

Month of study	Placebo (n=51)					EMPA (n=51)					Placebo vs. EMPA
	0	3	6	9	12	0	3	6	9	12	p value
FBG (mg/dL)	130.3 (25.7)	123.5 (22.9)	126.8 (24.8)	124.8 (32.9)	130.7 (26.2)	139.1 (36.1)	123.3 (21.9)*	122.9 (22.4)*	121.4 (22.5)*	122.1 (21.3)*	0.93
IRI (mU/mL)	9.1 (3.8)	8.7 (3.8)	9.2 (4.4)	9.5 (4.5)	10.3 (4.6)*	10.9 (6.2)	9.2 (4.4)*	8.4 (4.4)*	7.6 (3.3)*	6.5 (2.4)*	0.507
HOMA-IR	2.61 (1.26)	2.70 (1.38)	2.94 (1.54)*	3.06 (1.67)*	3.43 (1.92)*	3.56 (2.01)	2.87 (1.95)*	2.60 (1.60)*	2.27 (1.12)*	2.03 (1.01)*	0.0163^†^
HbA1c (%)	6.84 (0.85)	6.77 (0.81)	6.92 (0.88)	6.86 (0.93)	6.88 (0.76)	7.01 (1.1)	6.86 (0.82)*	6.88 (0.91)*	6.94 (0.96)*	6.90 (0.96)*	0.119
BMI	30.0 (4.4)	30.1 (4.3)	30.2 (4.4)	30.2 (4.6)	30.0 (3.7)	31.0 (4.8)	30.0 (4.9)*	29.5 (4.6)*	29.4 (4.5)*	29.4 (4.9)*	0.385
hsCRP (mg/L)	1.46 (1.4)	1.43 (1.56)	1.74 (1.87)	1.47 (1.66)	1.71 (1.64)	1.33 (1.0)	1.13 (0.73)	0.92 (0.68)*	0.73 (0.56)*	0.59 (0.42)*	0.00706^†^
LDL-C (mg/dL)	118.6 (25.1)	121.1 (25.6)	121.1 (23.8)	121.9 (24.4)	123.8 (25.8)	112.6 (27.9)	115.6 (27.3)	115.1 (23.4)	118.6 (20.0)	114.0 (19.7)	0.188
HDL-C (mg/dL)	55.1 (12.3)	54.7 (10.5)	55.4 (12.6)	54.5 (10.6)	53.3 (11.4)	55.3 (12.0)	57.8 (14.1)*	57.3 (12.9)*	58.8 (13.6)*	61.2 (15.2)*	0.222
TG (mg/dL)	129.4 (61.3)	139.8 (81.7)	142.6 (64.1)	139.9 (58.4)	153.5 (75.0)	157.8 (80.5)	130.4 (53.7)*	134.3 (54.7)*	127.6 (43.0)*	113.4 (54.6)*	0.553
RLP-C (mg/dL)	6.27 (3.96)	6.74 (5.65)	6.79 (4.28)	6.61 (4.13)	7.91 (5.57)	8.13 (5.02)	5.25 (3.17)*	5.26 (2.90)	4.87 (2.75)*	3.94 (2.10)*	0.029^†^
AST (IU/L)	27.2 (13.8)	27.8 (11.6)	25.8 (9.4)	26.9 (9.2)	27.1 (9.9)	28.7 (14.2)	25.6 (10.8)*	26.2 (10.1)	24.7 (8.6)	23.9 (9.4)*	0.583
ALT (IU/L)	33.2 (22.4)	35.3 (24.6)	34.4 (20.1)	34.3 (21.2)	35.7 (24.6)	37.0 (21.0)	32.0 (20.3)*	32.0 (19.6)*	31.0 (20.2)*	28.6 (20.1)*	0.6
γGTP (IU/L)	50.1 (36.1)	49.5 (33.2)	50.7 (34.3)	48.5 (28.7)	51.3 (30.5)	46.5 (32.4)	39.2 (23.8)*	41.0 (25.7)*	39.0 (24.6)*	35.1 (22.3)*	0.0558
eGFR (mL/min/1.73m2)	71.9 (18.6)	71.7 (19.5)	71.9 (19.0)	72.3 (17.5)	71.6 (19.1)	74.3 (16.3)	70.9 (16.4)*	71.2 (15.4)*	72.9 (17.4)	72.1 (16.6)	0.956
ACR (mg/gCr)	69.7 (289)	106.1 (425)	104.0 (467)	111.0 (514)	61.8 (149)	94.9 (244)	45.3 (78.3)	40.2 (68.7)	39.6 (65.1)	36.0 (62.4)	0.806
SBP (mmHg)	128.0 (15.6)	127.0 (17.4)	128.6 (16.6)	129.9 (15.5)	128.7 (15.6)	130.5 (21.2)	125.1 (14.8)*	126.3 (14.7)*	122.4 (13.7)*	121.0 (13.3)*	0.022^†^
DBP (mmHg)	77.5 (10.3)	75.8 (9.3)	77.1 (9.7)	77.2 (10.7)	75.5 (14.2)	78.1 (9.8)	71.9 (16.4)*	73.3 (11.0)*	72.2 (10.3)*	72.8 (10.5)*	0.0187^†^

Values are shown as means ± SD in parentheses

*BW* body weight, *BMI* body mass index, *FBG* fasting blood glucose, *HbA1c* hemoglobin A1c, *IRI* immuno reactive insulin, *HOMA-IR* homeostatic model assessment of insulin resistance, *LDL-C* LDL cholesterol, *HDL-C* HDL choresterol, *TG* triglyceride, *RLP-C* remnant-like particle cholesterol, *AST* aspartate aminotransferase, *ALT* alanine amino transferase, *γGTP* gamma-glutamyl transpeptidase, *eGFR* estimated glomerular filtration rate, *ACR* albumin-to-creatinine ratio, *hsCRP* highly sensitivity C-reactive protein, *SBP* systolic blood pressure, *DBP* diastolic blood pressure

*Intragroup comparison: p < 0 .05 (paired t test)

^†^Intergroup comparison for 12 months between placebo and EMPA: p < 0 .05 (repeated measures ANOVA)

We applied multiple linear regression analysis to identify factors associated with changes in hsCRP in the EMPA group. The objective variable comprised changes in hsCRP over a period of 12 months (log [hsCRP at 12 months] minus log [hsCRP at month 0]), and changes in FBG, IRI, BMI, HbA1c, AST, ALT, γ-GTP, LDL-cholesterol, HDL-cholesterol, RLP-C, eGFR, SBP, and DBP over 12 months were included as explanatory variables. The findings of these analyses (R^2^ = 0.9671, p = 4.709 × 10^−8^) revealed that changes in hsCRP were significantly associated with changes in HOMA-IR (t = 2.541, p = 0.0274), RLP-C (t = 2.528, p = 0.0281), SBP (t = − 2.324, p = 0.0403), HDL-cholesterol (t = − 2.411, p = 0.0346), and ALT (t = − 2.372, p = 0.0370) (Table 3).

**Table 3:** Association between changes in log[hsCRP] and various parameters coefficients

	Estimate	Std. error	t value	P
∆ACR (mg/gCr)	0.0006272	0.0003121	2.01	0.0697
∆BMI	− 0.004537	0.01105	− 0.411	0.6892
∆DBP (mmHg)	− 0.002671	0.002109	− 1.266	0.2316
∆eGFR (ml/min/1.73m2)	0.0002273	0.002345	0.097	0.9246
∆γGTP (IU/L)	− 0.001513	0.001401	− 1.08	0.3031
∆AST (IU/L)	0.003259	0.001935	1.684	0.1203
∆ALT (IU/L)	− 0.003582	0.00151	− 2.372	0.037*
∆HbA1c (%)	0.04275	0.02592	1.65	0.1273
∆HDL-C (mg/dL)	− 0.002794	0.001159	− 2.411	0.0346*
∆HOMA-IR	0.06453	0.0254	2.541	0.0274*
∆LDL (mg/dL)	0.0008015	0.0008732	0.918	0.3784
∆RLPC (mg/dL)	0.06442	0.02548	2.528	0.0281*
∆SBP (mmHg)	− 0.002882	0.00124	− 2.324	0.0403*
∆TG (mg/dL)	0.000000518	0.0003293	0.002	0.9988

R2 = 0.9855, p-value = 4.709 × 10^−8^

Multiple linear regression analysis was used to test the association between change in log[hsCRP] and changes in various parameters across 12 months in EMPA group

*hsCRP* high sensitivity C-reactive protein, *ACR* albumin-to-creatinine ratio, *BMI* body mass index, *eGFR* estimated glomerular filtration rate, *GTP*, gamma-glutamyl transpeptidase, *AST* aspartate aminotransferase, *ALT* alanine aminotransferase, *HbA1c* hemoglobin A1c, *HDL-C* HDL cholesterol, *LDL-C* LDL cholesterol, *TG* triglyceride, *RLP-C* remnant-like particle cholesterol, *SBP* systolic blood pressure, *DBP* diastolic blood pressure

*p < 0.05

## Discussion

This study showed that the SGLT2 inhibitor, EMPA, decreases hsCRP, lowers levels of remnant lipoproteins and ameliorates insulin resistance.

Insulin resistance is not a simple matter of deficient glucose uptake in response to insulin, but a multifaceted syndrome that significantly increases risk for CV disease [8]. How SGLT2 inhibitors improve insulin resistance has been examined using a glucose clamp procedure [9, 10]. However, the observation periods were relatively short and the patient cohorts were quite small because long-term assessment using this technique is difficult to implement. Here, we repeatedly measured HOMA-IR to evaluate changes in insulin resistance. We found that EMPA decreased HOMA-IR and significantly decreased hsCRP and RLP-C.

Three reports have described an association between hsCRP and insulin resistance [11–13]. The present study found a significant association between decreased hsCRP and ameliorated HOMA-IR, suggesting that improved insulin resistance results in anti-inflammatory effects.

Remnant lipoproteins are thought to be atherogenic. Remnant-like particle cholesterol (RLP-C) reflects amounts of various remnant lipoproteins in the blood. Increased levels of RLP-C are thought to comprise a significant and independent risk factor for coronary artery disease (CAD) and to predict future coronary events in patients with CAD and type 2 diabetes [14]. The present study found a significant correlation between changes with EMPA in RLP-C and in hsCRP. We recently found that EMPA decreases RLP-C levels in close association with ameliorated insulin resistance at 3 months in patients with diabetes and insulin resistance [15]. The present study found that this suppressive effect of EMPA on RLP-C continued for 12 months.

The CV benefits of EMPA might be due in part to anti-inflammatory effects as indicated by the 54% decrease in hsCRP in the present study. Arima et al. found that hsCRP levels were associated with future coronary heart disease events in a general Japanese population [16]. However, although hsCRP levels are far lower among Asians than other populations, the striking reduction in CV risk among Asians was similar to that in the overall population in the EMPA-REG OUTCOME trial [2]. Thus, a reduction in hsCRP within the lower range might confer a considerable benefit in CV risk reduction among Japanese patients with type 2 diabetes and insulin resistance.

This study has several limitations. We evaluated insulin resistance using HOMA-IR, which is not a good indicator under poor diabetes control. Another is that measuring HOMA-IR immediately after EMPA administration might not always reflect insulin resistance. This is because SGLT2 inhibitors work by inhibiting glucose reabsorption in the proximal tubules of the kidney, which results in increased urinary glucose excretion and decreased blood glucose levels. Thus, we excluded patients with poorly controlled diabetes from the present study. We also stopped EMPA administration for 72 h until SGLT2 inhibition became ineffective, then measured blood glucose and IRI in fasting blood samples to calculate HOMA-IR. Since the findings of linear regression analyses cannot define cause-and-effect between hsCRP and HOMA-IR and other parameters, further study is needed to clarify this issue.

## Conclusion

This study showed that the SGLT2 inhibitor, EMPA, ameliorated insulin resistance and might consequently decrease levels of hsCRP and remnant lipoproteins. We also showed that EMPA significantly decreased SBP and DBP. These results suggest that the CV benefits of EMPA might be driven at least in part, by anti-inflammatory effects that are particularly beneficial for patients with insulin resistance who might have cardiac dysfunction and/or vascular inflammation. This mechanism should cooperate with other EMPA-induced changes such as reduced blood pressure as shown herein and enhanced diuresis [17] to achieve the degree of cardioprotection revealed by the EMPA-REG OUTCOME trial.

## Abbreviations

ACR: albumin-to-creatinine ratio
ALT: alanine aminotransferase
AST: aspartate aminotransferase
BMI: body mass index
CAD: coronary artery disease
CV: cardiovascular
eGFR: estimated glomerular filtration rate
EMPA: empagliflozin
FBG: fasting blood glucose
HbA1c: hemoglobin A1c
HDL: high-density lipoprotein
HOMA-IR: homeostatic model assessment of insulin resistance
hsCRP: high sensitivity C-reactive protein
IRI: immunoreactive insulin
LDL: low-density lipoprotein
RLP-C: remnant-like particle cholesterol
SGLT2: sodium–glucose cotransporter 2 inhibitor
γGTP: gamma-glutamyl transpeptidase
